# Introduction to Editorial Board Members: Prof Nicholas A. Peppas

**DOI:** 10.1002/btm2.10011

**Published:** 2016-06-23

**Authors:** Samir Mitragotri

**Affiliations:** ^1^ Dept. of Chemical Engineering Center for Bioengineering University of California Santa Barbara CA 93106

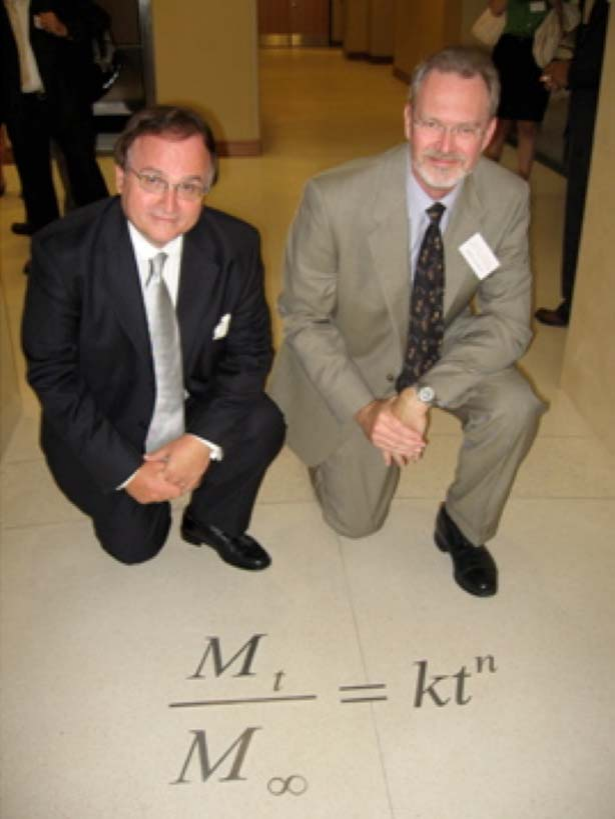

On the launch of this inaugural issue of AIChE's and SBE's *Bioengineering & Translational Medicine*, I must express my deep gratitude to the members of our Editorial Advisory Board. Their expertise and experience provide a strong foundation supporting the broad range of bioengineering topics that will be covered in the journal. To highlight this strength, we plan to feature one of our distinguished Editorial Advisory Board members in each issue. In this inaugural issue, we are proud to feature Prof Nicholas A Peppas.

Nicholas Peppas holds a Cockrell Family Regents Chaired Professorship in Engineering, Medicine and Pharmacy at the University of Texas at Austin, with appointments in the departments of Chemical Engineering and Biomedical Engineering, in Surgery in the Dell Medical School, and in the College of Pharmacy. He received his Diploma in Engineering from the National Technical University in Athens and an Sc.D. from the Massachusetts Institute of Technology in Chemical Engineering. He has also received numerous honorary doctorates, including those from the University of Ghent (Belgium), the University of Parma (Italy), the University of Ljublijana (Slovenia), the University of Athens (Greece), the University of Patras (Greece), and an honorary professorship from the Sichuan University (China).

Professor Peppas' research has made a transformative impact on drug delivery, which has leaped to the forefront of science and technology. In particular, Prof Peppas' research on mathematical models and novel biomaterials has yielded not only new insights into drug release mechanisms and new technologies to control them, but has also provided a new paradigm for research conducted at the interface of medicine and engineering. His early studies on drug diffusion in polymeric systems provided the much needed quantitative tools to describe drug release,^1^ an equation that is now commonly known as Korsemeyer‐Peppas model. This particular study established a number of key fundamental principles of drug release from porous matrices. Specifically, swellable porous matrices exhibit complex release behavior arising from swelling‐induced structural changes of the matrix. This pioneering work provided numerous insights into drug release mechanisms, including dependence on drug size, the role of matrix composition, and the role of matrix swelling. Further, this study provided the vision that, if the matrix materials and additives are chosen carefully, it should be possible to control the drug release to a desired rate. The lessons learned from these studies have fueled groundbreaking fundamental as well as translational advances in the field of hydrogel‐based drug delivery systems. Today, hydrogel‐based drug delivery systems are used in various applications including wound dressings, contact lenses, mucosal delivery systems, topical formulations, and depot injections, among others. The impact of this paper is also clear from the fact that this equation has been cited more than 2,700 times to date. Overall, Dr Peppas' research has been cited over 84,000 times with an h‐index of 140, making him one of most cited researchers in the world across all disciplines.

In 1987, Prof Peppas published two seminal papers in the *Journal of Controlled Release* on mathematical models of solute diffusion.^2^, ^3^ These publications introduced equations to describe Fickian and non‐Fickian diffusional release from nonswellable polymeric delivery systems of various geometries. These models provided a simple‐to‐use, yet accurate, equation to describe drug release and these two publications remain the most cited original research articles in the *Journal of Controlled Release* in its 30‐plus year history.

Responsive hydrogels represent another area where Dr Peppas' work has made a pioneering and transformative impact, especially for applications in diabetes treatment.^4^, ^5^, ^6^, ^7^, ^8^ Hydrogels that respond to pH, glucose, or other stimuli have opened new opportunities in designing materials whose key properties, especially drug release, can be dynamically tuned. pH‐responsive poly(methacrylic‐ethylene glycol) hydrogels that swell in response to high pH provide materials that exhibit a collapsed state in the acidic environment of the stomach and, thus, protect encapsulated insulin from the gastric enzymes. Upon exposure to high pH of the intestine, on the other hand, the same hydrogel can swell and release insulin to facilitate oral absorption. These smart hydrogels have offered an alternative to injections, which currently represents the most common mode of insulin administration to diabetic patients despite their severe limitations.

Encapsulation of glucose oxidase in hydrogel adds another layer of “smartness” to gel‐based systems.^5^ Glucose oxidase‐containing gels exhibit a higher rate of swelling, thus offering insulin release that depends on local glucose concentrations. Such responsive hydrogels have been efficacious in insulin delivery, producing a strong, dose‐dependent and long‐lasting hypoglycemic effect in diabetic rats. In recent studies, these hydrogels have also been used to deliver macromolecules such as siRNA for the treatment of gastrointestinal inflammatory conditions.^9^ Professor Peppas' work has impacted many additional areas of bioengineering, including mechanisms of transport across biomembranes, analysis of tissue‐polymer interactions, and transport properties of complex polymer systems.

Professor Peppas has been recognized with numerous awards, including elections to the National Academy of Engineering (NAE), National Academy of Medicine (NAM), National Academy of Inventors (NAI), National Academy of France, Royal Academy of Spain and the Academy of Athens. He is also an elected fellow of the American Institute of Medical and Biological Engineering (AIMBE), Biomedical Engineering Society (BMES), American Association of Pharmaceutical Scientists (AAPS), American Association of Advancement of Science (AAAS), Society for Biomaterials (SFB), among others. Professor Peppas has been recognized by the NAE Founders Award, the Giulio Natta Medal, the Acta Biomaterialia Gold Medal, AIMBE's Pierre Galletti Award, and the Maurice Marie Janot Award in Pharmaceutical Sciences, among others. His teaching accomplishments have been recognized through the Benjamin Garver Lamme Excellence in Engineering Education Award from the American Society for Engineering Education. In 2016, Prof Peppas was included in the 100 Power List by the medicinemaker, comprising the most influential individuals making medicines.

AIChE and SBE have been particularly proud to recognize Prof Peppas with several of their distinguished awards, including Materials Engineering and Sciences Award (1984); Food, Pharmaceuticals, and Bioengineering Award (1992); Nanoscale Science and Engineering Award (2014), Founders Award for Outstanding Contributions to Chemical Engineering (2008); William H. Walker Award (2006); Jay Bailey Award (2006); and Institute Lecturer (2007). In 2008, Prof Peppas was listed among the “One Hundred Chemical Engineers of the Modern Era by AIChE” on the occasion of the Institute's centennial.

As towering as these accomplishments and recognitions are, if we ask Prof Peppas, he would say that his proudest professional achievements are his students. To date, he has supervised more than 875 researchers, including 105 PhDs, over half of whom are now professors at various Universities. His students are advancing various frontiers of bioengineering and biotechnology in their own ways in academia as well as industry.

Over the years, Prof Peppas has played a central role in advancing the cause of bioengineering in various organizations, especially in AIChE. He was director of AIChE (1999–2002); chair of the AIChE's Materials Engineering and Sciences Division (1988–1990); director of AIChE's Food, Pharmaceutical and Bioengineering Division (1994–1997); and founder of AIChE's Bionanotechnology programming area. Even outside AIChE, Prof Peppas has been extremely active in various professional organizations. He was director of the BMES (2008–2011), Chair of the College of Fellows of AIMBE (2006–2007), President of the SFB (2003–2004), president of the Controlled Release Society (1987–1988), and chair of the Engineering section of AAAS (2014–2015). He is presently the President of the International Union of Societies for Biomaterials Sciences and Engineering (2008–2016). Among many of his editorial responsibilities, he was Associate Editor of the *AIChE Journal* (2009–2012). Professor Peppas' pioneering research, along with his active and inspiring leadership in various professional organizations, has advanced the visibility of drug delivery in the scientific landscape, and has inspired young professionals to follow the suit.

On behalf of AIChE and SBE, I thank Prof Peppas for his strong support in the launch of *Bioengineering & Translational Medicine* and look forward to his continued support.

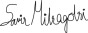

Samir Mitragotri Editor‐in‐Chief *Dept. of Chemical Engineering Center for Bioengineering University of California Santa Barbara, CA 93106 Email:* samir@engr.ucsb.edu

## References

[1] Korsemeyer RW , Gurny R , Doelker E , Buri P , Peppas NA. Mechanisms of solute release from porous hydrophilic polymers. Int J Pharm. 1983;15:25–35.

[2] Ritger PL , Peppas NA. A simple equation for description of solute release II. Fickian and anomalous release from swellable devices. J Control Release. 1987;5:37–42. 25356469

[3] Ritger PL , Peppas NA. A simple equation for description of solute release I. Fickian and non‐Fickian release from non‐swellable devices in the form of slabs, spheres, cylinders or discs. J Control Release. 1987;5:23–36 25356469

[4] Lowman AM , Morishita M , Kajita M , Nagai T , Peppas NA. Oral delivery of insulin using pH‐responsive complexation gels. J Pharm Sci. 1999;88(9):933–937. 10479357 10.1021/js980337n

[5] Podual K , Doyle FJ III , Peppas NA. Dynamic behavior of glucose‐oxidase‐containing microparticles of poly(ethylene glycol)‐grafted cationic hydrogels in an environment of changing pH. Biomaterials. 2000;21:1439–1450. 10872773 10.1016/s0142-9612(00)00020-x

[6] Torres‐Lugo M , García M , Record R , Peppas NA. pH‐Sensitive hydrogels as gastrointestinal tract absorption enhancers: transport mechanisms of salmon calcitonin and other model molecules using the Caco‐2 cell model. Biotechnol Prog. 2002;18:612–616. 12052080 10.1021/bp0101379 PMC4467728

[7] Serra L , Doménech J , Peppas NA. Drug transport mechanisms and release kinetics from molecularly designed poly(acrylic acid‐*g*‐ethylene glycol)hydrogels. Biomaterials. 2006;27(31):5440–5451. 16828864 10.1016/j.biomaterials.2006.06.011

[8] Kryscio DR , Peppas NA. Mimicking biological delivery through feedback‐controlled, recognitive drug release systems based on molecular imprinting methods. AIChE J. 2009;55:1311–1324. 26500352 10.1002/aic.11779 PMC4613793

[9] Knipe JM , Strong LE , Peppas NA. Enzyme‐ and pH responsive microencapsulated nanogels for oral delivery of siRNA to induce TNF‐α knockdown in the intestine. Biomacromolecules. 2016;17(3):788–797. 26813877 10.1021/acs.biomac.5b01518

